# Supplementary material for: MetaQTL: a package of new computational methods for the meta-analysis of QTL mapping experiments
Source: BMC Bioinformatics. 2007 Feb 8;8:49. doi: 10.1186/1471-2105-8-49 (PMC1808479; doi:10.1186/1471-2105-8-49)
Supplement: Additional File 7 — MetaQTL Package : jar file and tutorial. This Zip archive contains both the MetaQTL JAR file and the files of the tutorial. [file 1471-2105-8-49-S7.zip › org.inra.metaqtl/doc/org/metaqtl/class-use/MetaQtlData.html]

Uses of Class org.metaqtl.MetaQtlData


|  |  |  |
| --- | --- | --- |
| **Overview** \| **Package** \| **Class** \| **Use** \| **Tree** \| **Deprecated** \| **Index** \| **Help** | |  |
| PREV   NEXT | **FRAMES**    **NO FRAMES**     **All Classes** |  |


---


## **Uses of Class org.metaqtl.MetaQtlData**

| Packages that use MetaQtlData | |
| --- | --- |
| **org.metaqtl.adapter** |  |
| **org.metaqtl.algo** |  |

Uses of MetaQtlData in org.metaqtl.adapter

| Methods in org.metaqtl.adapter with parameters of type MetaQtlData | |
| --- | --- |
| `static QtlPartition` | `QtlPartitionAdapter.adapt(MetaQtlData qtlData)`             Adapt the given `MetaQtlData` into a `QtlPartition` and returns it. |

Uses of MetaQtlData in org.metaqtl.algo

| Methods in org.metaqtl.algo with parameters of type MetaQtlData | |
| --- | --- |
| `MetaQtlResult[]` | `QtlTreeAlgorithm.buildQtlTree(MetaQtlData data)`             For the given data points this method build the tree which represents the hierarchical clustering. |
| `MetaQtlResult[]` | `QtlClustAlgorithm.doQtlClustering(MetaQtlData data)`             For a given data set `data` performs the clustering on each trait group and returns the results as an array of `MetaQtlResult` objects. |

---


|  |  |  |
| --- | --- | --- |
| **Overview** \| **Package** \| **Class** \| **Use** \| **Tree** \| **Deprecated** \| **Index** \| **Help** | |  |
| PREV   NEXT | **FRAMES**    **NO FRAMES**     **All Classes** |  |


---
